# Objective Measures for the Assessment of Post-Operative Pain in *Bos indicus* Bull Calves Following Castration

**DOI:** 10.3390/ani7100076

**Published:** 2017-09-28

**Authors:** Gabrielle C. Musk, Stine Jacobsen, Timothy H. Hyndman, Heidi S. Lehmann, S. Jonathon Tuke, Teresa Collins, Karina B. Gleerup, Craig B. Johnson, Michael Laurence

**Affiliations:** 1 College of Veterinary Medicine, School of Veterinary and Life Sciences, Murdoch University, Murdoch, WA 6150, Australia; t.hyndman@murdoch.edu.au (T.H.H.); H.Lehmann@massey.ac.nz (H.S.L.); T.Collins@murdoch.edu.au (T.C.); m.laurence@murdoch.edu.au (M.L.); 2 Department of Large Animal Sciences, University of Copenhagen, DK-2630 Copenhagen, Denmark; stj@sund.ku.dk (S.J.); kbg@sund.ku.dk (K.B.J.); 3 School of Mathematics, University of Adelaide, Adelaide, SA 5005, Australia; simon.tuke@adelaide.edu.au; 4 Institute of Veterinary, Animal and Biomedical Sciences, Massey University, Palmerston North 4442, New Zealand; c.b.johnson@massey.ac.nz

**Keywords:** analgesia, husbandry, Brahman, Australia, welfare

## Abstract

**Simple Summary:**

Surgical castration of cattle is a common husbandry procedure, and although this procedure is known to cause pain in cattle and other species, in some countries it is often performed without anaesthesia or analgesia. Society is increasingly aware of this animal welfare issue and it is creating pressure to drive research into animal welfare science with the aim of identifying practical and economical approaches to pain management in livestock. To effectively manage pain, a pain assessment must be performed. Pain assessment methods are often subjective and therefore influenced by the observer. Ideally, objective assessments that generate consistent and repeatable results between observers should be identified. *Bos indicus* bull calves were divided into four groups: no castration (NC, *n* = 6); castration with pre-operative local anaesthetic (CL *n* = 12); castration with pre-operative anti-inflammatory medication (CM, *n* = 12); and, castration without pain relief (C, *n* = 12). A range of objective assessments was performed: bodyweight measurements, activity, and rest levels, and four different compounds in the blood. The results of this study suggest that animals rest for longer periods after the pre-operative administration of anti-inflammatory medication. The other objective assessments measured in this study were not able to consistently differentiate between treatment groups. These findings emphasise the need for alternative quantifiable and objective indicators of pain in *Bos indicus* bull calves.

**Abstract:**

The aim of the study was to assess pain in *Bos indicus* bull calves following surgical castration. Forty-two animals were randomised to four groups: no castration (NC, *n* = 6); castration with pre-operative lidocaine (CL, *n* = 12); castration with pre-operative meloxicam (CM, *n* = 12); and, castration alone (C, *n* = 12). Bodyweight was measured regularly and pedometers provided data on activity and rest from day −7 (7 days prior to surgery) to 13. Blood was collected for the measurement of serum amyloid A (SAA), haptoglobin, fibrinogen, and iron on days 0, 3 and 6. Bodyweight and pedometry data were analysed with a mixed effect model. The blood results were analysed with repeated measure one-way analysis of variance (ANOVA). There was no treatment effect on bodyweight or activity. The duration of rest was greatest in the CM group and lowest in the C group. There was a significant increase in the concentrations of SAA, haptoglobin, and fibrinogen in all of the groups from day 0 to 3. Iron concentrations were not different at the time points it was measured. The results of this study suggest that animals rest for longer periods after the pre-operative administration of meloxicam. The other objective assessments measured in this study were not able to consistently differentiate between treatment groups.

## 1. Introduction

Surgical castration of cattle is a common husbandry procedure, and although this procedure is known to cause pain in cattle, in some countries it is often performed without anaesthesia or analgesia [1]. Society is increasingly aware of this animal welfare issue and is creating pressure to drive research into animal welfare science with the aim of identifying practical and economical approaches to pain management in livestock [2]. A challenge in recommending an analgesic therapy for a species undergoing a particular procedure is that the efficacy of such a strategy can only be determined if pain can be assessed reliably. Furthermore, the local legislative framework and financial cost of an analgesic strategy needs to be considered. Consequently, ongoing investigation into both analgesic strategies and methods of pain assessment are required in the context of primary production. *Bos indicus* cattle undergoing castration are no exception to this approach, especially as they are the predominant species of cattle farmed in the north of Australia [3]. 

By its very nature, pain is a complex sensory and emotional experience [4]. This complexity means that it is a challenge to find methods for pain assessment that are reliable, repeatable and consistent between observers [5]. The ideal method of pain assessment will be simple to use, objective, and quantifiable [6]. Objective assessments in this context are those that are measurable and consistent when different instruments are used to attain the measurement. For *Bos indicus* cattle undergoing castration, both objective and subjective assessments of pain and stress have been utilised [7,8,9,10]. Objective assessments of pain or inflammation include evaluation of changes in blood concentrations of circulating cortisol, haptoglobin, creatinine kinase, total protein, and packed cell volume, as well as nociceptive threshold testing, and changes in bodyweight and activity (using pedometry) [8,9,10]. Other assessments in *Bos indicus* cattle following castration are based almost exclusively on the observation and the interpretation of behaviour, which are more challenging and time consuming to quantify, but may more accurately capture the animals’ response to the environment [11,12]. It is important to acknowledge that *Bos indicus* cattle are more habituated to stockpeople, are more excitable [13] and behaviourally and physiologically more reactive to handling than *Bos taurus* [14]. Consequently, species specific investigations are justified.

It has previously been demonstrated that bodyweight and pedometry could serve as objective measures to differentiate analgesic treatment groups in *Bos indicus* cattle following castration [8]. Bodyweight changes have implications for the profitability of livestock production and may influence the economic decision-making process if analgesia strategies are to be incorporated [10]. Also, bodyweight change is used as a welfare indicator, since pain and distress may reduce feed intake (in *Bos taurus* cattle), and in turn limit weight gain in growth periods or cause weight loss [15]. Pedometry assesses animal movement and has been used as a surrogate marker for a range of behavioural patterns for animals before and after a procedure. Various parameters can be determined from the raw pedometry data, including the average number of steps per hour, and the duration and frequency of rest periods [8]. Commercially available pedometers are small and non-invasive and are unlikely to have an influence on natural exercise patterns [16]. Furthermore, pedometers provide continuous and individual animal data, which are difficult to obtain by other means [16]. 

Acute phase proteins (APPs) such as SAA, haptoglobin, and fibrinogen are synthesised in the liver in response to cytokines involved with inflammation and infection [17]. Blood concentrations of these three proteins are known to increase during an acute phase reaction in cattle by up to ten fold (SAA), greater than ten-fold (haptoglobin), and 50–100% (fibrinogen) [17]. Iron is a negative acute phase reactant, meaning that blood concentrations are expected to decrease in response to tissue injury and inflammation [18,19].

Lidocaine is a local anaesthetic drug with a fast onset of action and an intermediate duration of action [20]. The mechanism of action of local anaesthetic drugs is blockade of sodium channels within nerve cells, which inhibits the conduction and transmission of nociception [21]. Lidocaine is commonly incorporated into a protocol for castration by veterinary practitioners working with cattle [1,22]. Meloxicam is a non-steroidal anti-inflammatory drug that is one of the most potent inhibitors of the cyclooxygenase-2 enzyme [23]. Meloxicam has also been used to provide analgesia for cattle undergoing castration [21] and following subcutaneous injection minimum therapeutic plasma concentrations are achieved after 30 min in cattle [23].

The aim of this study was to apply objective measures to differentiate treatment groups following surgical castration with lidocaine (for local anaesthesia), meloxicam (for systemic analgesia), or without analgesia. The animals in this study were all anaesthetised with halothane during castration. The objective measures included in this study were bodyweight, pedometry (activity) and the blood concentrations of three acute phase proteins (APPs), and iron. It was hypothesised that animals treated with either local anaesthesia with lidocaine or subcutaneous meloxicam prior to castration would continue to gain weight at a rate consistent with their age; be more active; demonstrate an increase in circulating SAA, haptoglobin and fibrinogen; and, a simultaneous decrease in circulating iron concentration following castration, when compared to non-treated castrated controls.

## 2. Materials and Methods 

The study was approved by the Animal Ethics Committee of Murdoch University (R2730/15), in accordance with the Code of Practice for the Care and Use of Animals for Scientific Purposes [24]. Forty-two *Bos indicus* bull calves aged six-to-eight months of age were sourced from an extensive cattle station in the northwest of Australia. The animals were transported to the Murdoch University teaching farm in Western Australia two weeks prior to the study. 

On arrival at the university farm, the animals were randomly allocated a study identification number. The animals were held in a 1.1 ha paddock with *ad libitum* access to Kikuyu pasture, oaten hay and water and were fed a complete pelleted ration at approximately 3% of bodyweight each morning (EasyBeef pellets, Milne AgriGroup, Pty Ltd., Perth, Western Australia, Australia). During the two-week acclimatisation period, the bulls were exposed to human interaction but specific training for the procedures required for general anaesthesia was not undertaken.

Cattle were randomised to four study groups: no castration control group (NC, *n* = 6); castration with lidocaine (CL, *n* = 12); castration with meloxicam (CM, *n* = 12); and, castration without analgesia (C, *n* = 12). Group allocation was via block randomisation to ensure that the last animal to be castrated on each day was equally represented in the three study groups. Four animals were castrated each experimental day. The numbers that were chosen reflected a previous study in cattle assessing EEG changes following noxious stimuli [25] where treatment groups of ten were assessed. Every animal underwent general anaesthesia with halothane as previously described [26]. On the day of anaesthesia, the animals were transported approximately 1.5 km in a cattle truck from the farm paddock to a covered shed with an adjacent paddock for recovery. Pelleted food was not provided to these animals on the morning of anaesthesia and surgery. 

Animals in the NC group were anaesthetised for approximately 45 min and were not subjected to any procedures beyond instrumentation for physiological monitoring during anaesthesia [26]. In the CL group, lidocaine (260 mg, Ilium Lignocaine 20, 20 mg/mL, Troy Laboratories, Glendenning, Australia) was injected into the testicles and subcutaneous tissue of the scrotum five minutes prior to castration. Each injection of lidocaine was divided so that approximately 5.5 mL of drug was injected into each testicle and the remaining 2 mL was injected subcutaneously into the scrotal skin at the incision site, distally on the scrotum. In the CM group, meloxicam (0.5 mg/kg, Ilium Meloxicam 20, 20 mg/mL, Troy Laboratories, Australia) was injected subcutaneously on the right lateral neck 30 min prior to castration (at the start of anaesthesia). Analgesia was not administered to animals in the C group. Surgical castration was performed during general anaesthesia by a single experienced veterinary surgeon using an open technique and the wounds were left to heal by secondary intention [27]. Briefly, the skin and tunica vaginalis were sharply incised, the connective tissue surrounding the testicle was dissected bluntly, after which continuous traction was placed on the spermatic cord until a rupture occurred. The surgical wound was then left open.

For post-operative pain assessment, bodyweight, pedometry (activity, total rest time, and duration of rests), and systemic inflammatory markers were measured. Bodyweight was measured on an in-race electronic scale (Gallagher Animal Management, Australia). The scales were calibrated at the start of the study. Bodyweight was measured prior to anaesthesia on day −7 (7 days prior to surgery), on the day of anaesthesia (day 0) and on post-procedure days 6 and 10 in all of the groups, and again on day 13 for groups CL, CM, and C. A pedometer was fitted with a broad nylon strap above the fetlock of the left hind limb to each animal as previously described (Afi2go, Afimilk Ltd., Kibbutz Afikim 1514800, Israel) [8]. Pedometry data were communicated wirelessly to a base unit mounted in the paddock. The range of the pedometers from the base unit was 400 m, exceeding the longest dimension of the paddock. The base unit had a 12 h recall function (Afi2go, Afimilk Ltd., Kibbutz Afikim 1514800, Israel). Activity (steps/hour), total rest time (total minutes/day), and duration of rests (average time (minutes) of each rest period) were calculated daily from data collected from the pedometer using proprietary software (Afifarm, Afimilk Ltd., Kibbutz Afikim 1514800, Israel). Pedometry data were recorded continuously from seven days prior to anaesthesia (day −7) to 13 days after anaesthesia (day 13).

For measurement of systemic inflammatory markers, 15 mL of blood was collected from the jugular vein during anaesthesia (day 0), and on post-procedure days 3 and 6 while the animal was restrained in a cattle crush. The blood samples were collected into tubes containing citrate (3.2% sodium citrate, mix ratio: 1 part citrate to 9 parts blood) and into plain serum tubes. The tubes were centrifuged, and plasma (citrate blood tubes) or serum (plain blood tubes) was separated within six hours of collection. Plasma and serum samples were stored in polypropylene cryovials at −20 °C for five months. Samples were then shipped on dry ice to the Veterinary Diagnostic Laboratory, University of Copenhagen, Denmark, for analysis.

The concentration of serum amyloid A (SAA) in serum was determined using a commercially available sandwich ELISA assay (Phase Range SAA assay, Tridelta Development Ltd., Maynooth, Ireland), as previously described [28]. The samples and standards were tested in duplicate in a 1:500 dilution as per the manufacturer’s instructions. Samples with an optical density outside of the range of the standard curve were further diluted and re-analysed in a 1:2000 dilution. The lower limit of detection was 0.5 mg/L. Optical density at 450 nm was read and corrected for background optical density at 650 nm using a dedicated ELISA reader (Thermo Multiskan Ex spectrophotometer, Thermo Scientific, Waltham, MA, USA). Haptoglobin was measured in serum with a commercially available assay (Phase Range Haptoglobin assay, Tridelta Development Ltd., Kildare, Ireland) according to the manufacturer’s instruction using an ADVIA 1800 (ADVIA 1800 Chemistry System, Siemens Health Care Diagnostics Inc., Tarrydown, NY, USA), as previously described [29]. Fibrinogen concentrations were measured in citrate-stabilised plasma by the Clauss method in an automated coagulometric analyser (ACL 9000, Instrumentation Laboratory, Barcelona, Spain). Serum iron concentrations were determined by colorimetric spectrophotometry (ADVIA 1650; Bayer A/S, Lyngby, Denmark).

Animals were monitored for at least two weeks in the post-operative period and if any animal was considered to be experiencing mild to moderate pain, independent veterinary advice was sought. Assessment of post-operative pain was based upon observation of locomotion, interactive behaviour, activity, appetite, and exhibition of unusual behaviours such as licking the surgical wound and arching the back [12]. Rescue analgesia was morphine (0.1–0.3 mg/kg) by intramuscular (IM) injection and/or meloxicam (0.5 mg/kg) by subcutaneous (SC) injection. 

### Statistical Analyses 

Data from the bodyweight and pedometry variables were analysed with a mixed effect model (R Core Team R: A Language and Environment for Statistical Computing. Vienna, Austria: R Foundation for Statistical Computing. https://www.R-project.org/2017). For bodyweight, activity, total rest time, and duration of rests, a linear model was fitted with day, pre-treatment measure, and treatment as predictors. Model selection was performed by starting with a full model up to and including two-way interaction; followed by using likelihood ratio tests with a cutoff of <0.05 to find the most parsimonious model. To account for the longitudinal nature of the data, a random intercept was included in the models. The models were fitted using the nlme package [30]. The final predictors for bodyweight were pre-treatment bodyweight and day. The final predictors for activity, total rest time, and duration of rests were: pre-treatment activity and day; pre-treatment rest, day, treatment, and interaction between day and treatment; pre-treatment duration of rests, day and treatment, respectively. The pre-treatment level was the mean of the response variable for each bull before any treatment was given. The day was treated as a categorical random variable. For each model, the random intercept for each bull fitted the data the best. Analyses of the measured variables in blood were repeated measure one-way analysis of variance (GraphPad Prism 7, GraphPad Software, La Jolla, CA, USA; GraphPad 2016, GraphPad Software, La Jolla, CA, USA). Data are presented as mean (±SD). Significance was defined at *p* < 0.05.

## 3. Results

Recovery from anaesthesia was uneventful in all of the animals except for one in the NC group. This animal sustained a soft tissue injury of the left hind limb during recovery from anaesthesia. Analgesia was administered (morphine 0.1 mg/kg IM and meloxicam 0.5 mg/kg SC and the lameness resolved within 12 h. Data from this animal were subsequently excluded from analyses. No animals that were castrated required rescue analgesia.

**Figure 1:**
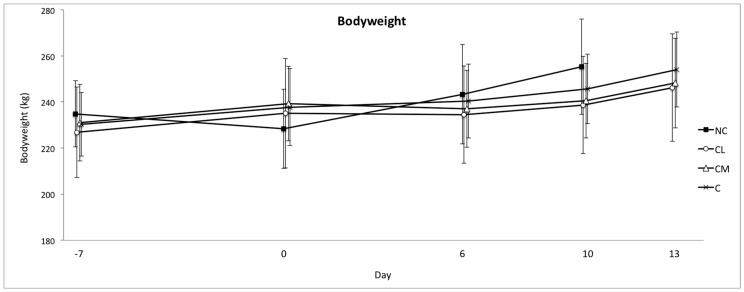
Mean (±SD) bodyweight (kg) of *Bos indicus* bull calves from −7 (7 days prior to surgery) to 13 days after surgery. NC = no castration, CL = castration with lidocaine, CM = castration with meloxicam, C = castration without analgesia.

**Figure 2:**
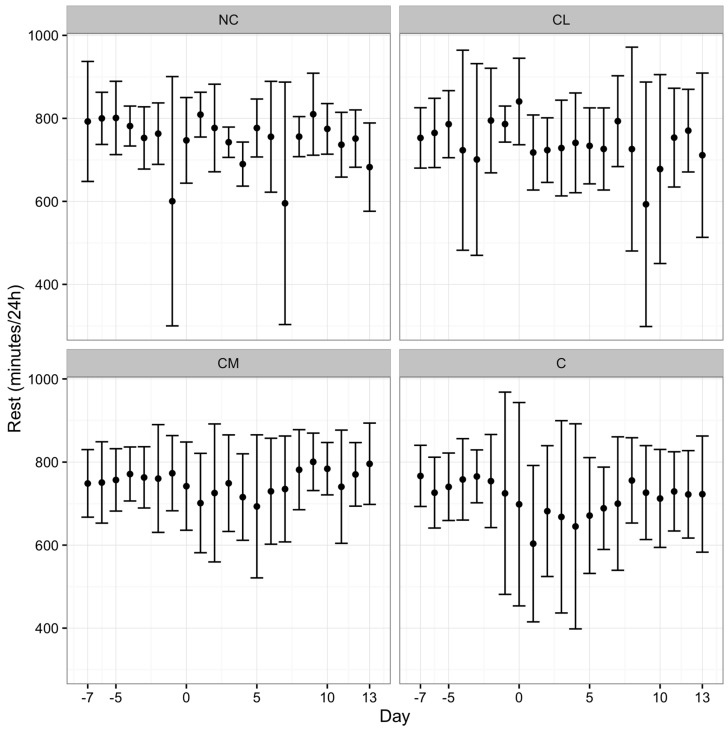
Mean (±SD) rest (min/24 h) from −7 days prior to surgery to 13 days after surgery. CL = castration with lidocaine, CM = castration with meloxicam, C = castration, NC = no castration.

**Figure 3:**
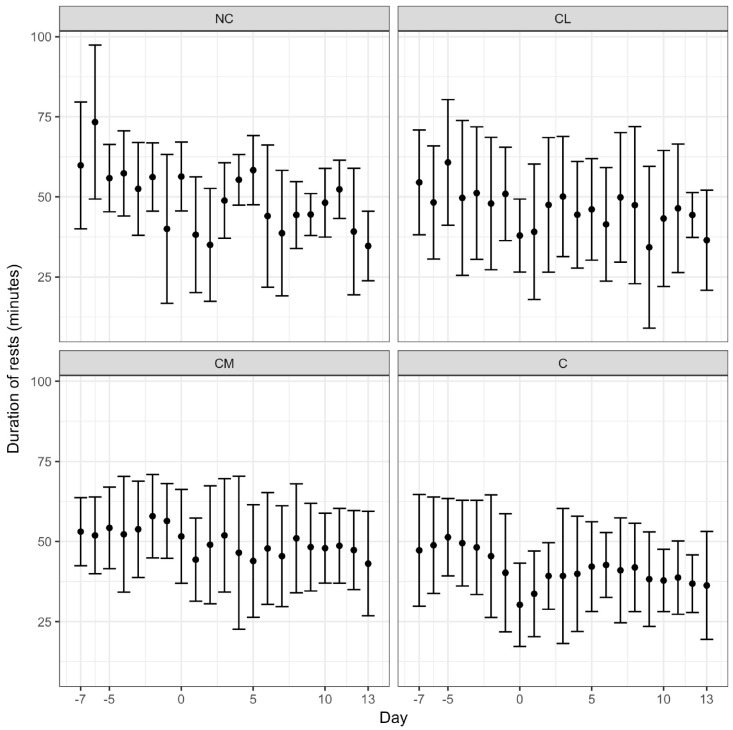
Mean (±SD) duration of rests (average time spent resting (minutes)) from −7 days prior to surgery to 13 days after surgery. CL = castration with lidocaine, CM = castration with meloxicam, C = castration, NC = no castration.

All of the cattle gained weight during the study (Figure 1). The model for bodyweight change could not be fitted with day and treatment interaction terms (the NC group was excluded from the model). There was no treatment effect on bodyweight over the course of the study. The pedometry data generated three parameters: activity (steps/h), total rest time (min/24 h) and duration of rests (average time (min) of each rest period). The NC group was maintained in the model. There was no treatment effect for activity. For rest, there was an effect of treatment and day whereby the duration of rest over the study was greatest in the CM group and lowest in the C group, *p* < 0.0001 (Figure 2). For the duration of rests, there was a treatment effect in that this variable was longest in the CM group and shortest in the C group, *p* < 0.0001 (Figure 3). Finally, those animals that had longer durations of rest prior to anaesthesia maintained the pattern of taking longer rests after anaesthesia.

**Figure 4:**
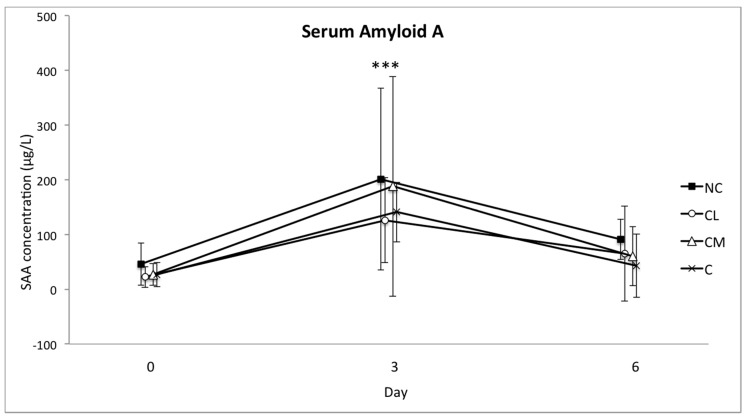
Mean (±SD) concentration of Serum Amyloid A (SAA) in serum (μg/L) of Bos indicus bull calves before and after surgical castration. NC = no castration, CL = castration with lidocaine, CM = castration with meloxicam, C = castration without analgesia. *** *p* < 0.001 difference in all groups from day 0 to day 3.

**Figure 5:**
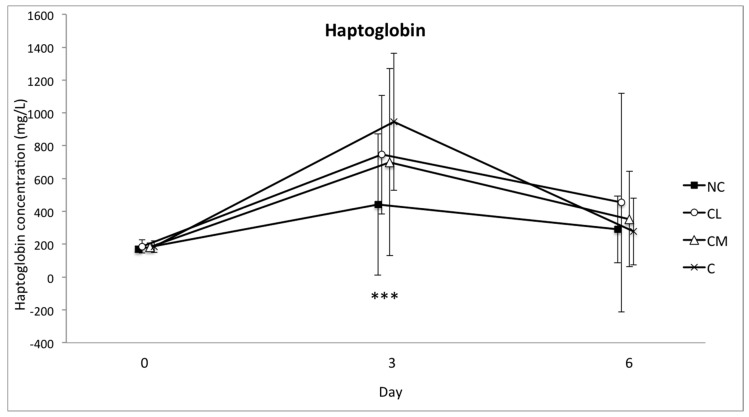
Mean (±SD) concentration of haptoglobin in serum (mg/L) of Bos indicus bull calves before and after surgical castration. NC = no castration, CL = castration with lidocaine, CM = castration with meloxicam, C = castration without analgesia. *** *p* < 0.001 difference in all groups from day 0 to day 3.

**Figure 6:**
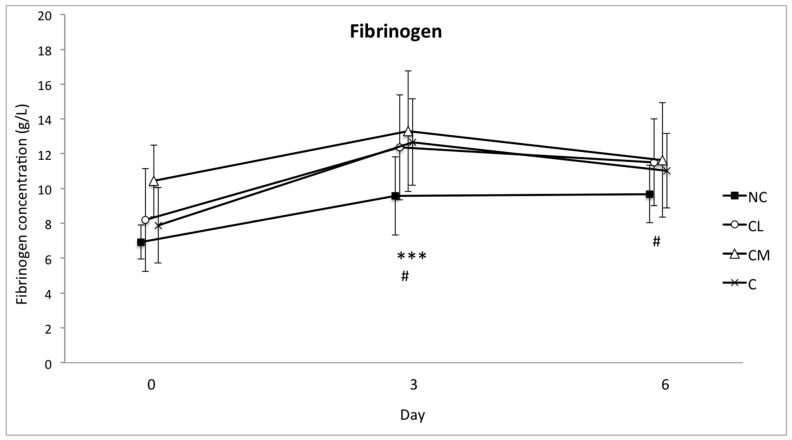
Mean (±SD) concentration of fibrinogen in plasma (g/L) of Bos indicus bull calves before and after surgical castration. NC = no castration, CL = castration with lidocaine, CM = castration with meloxicam, C = castration without analgesia. *** *p* < 0.001 difference in all groups from day 0 to day 3 and between groups on day 3. # *p* = 0.0003 the plasma concentration of fibrinogen was lowest in the NC group on days 3 and 6.

**Figure 7:**
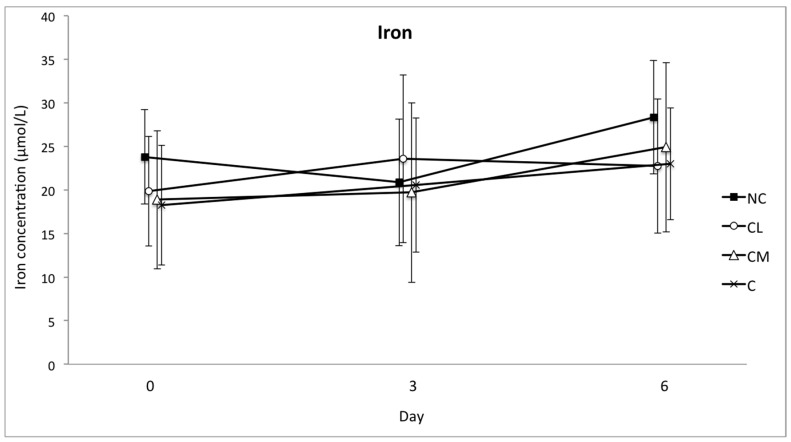
Mean (±SD) concentration of iron in serum (μmol/L) of Bos indicus bull calves before and after surgical castration. NC = no castration, CL = castration with lidocaine, CM = castration with meloxicam, C = castration without analgesia.

The mean serum concentrations of SAA and haptoglobin increased in all of the groups from the baseline (day 0) to day 3. This increase was significant (*p* < 0.001), but there was no difference between the groups (Figure 4 and Figure 5). The concentration of fibrinogen increased significantly in all of the groups from the baseline (day 0) to day 3 (*p* < 0.0001). The magnitude of this change was different between the groups (*p* = 0.0003), with the lowest plasma concentration of fibrinogen in the NC group on days 3 and 6 (Figure 6). The serum concentration of iron was not different between the groups or within the groups over time (Figure 7).

## 4. Discussion

The aim of this study was to apply objective measures to differentiate two analgesic and one no-analgesic treatment group in *Bos indicus* bull calves following surgical castration performed during halothane anaesthesia. The analgesic treatment groups were lidocaine or meloxicam, and the objective measures were bodyweight, pedometry (activity), and the blood concentrations of three acute phase proteins and iron. The differences that were observed between the treatment groups across the objective measures included in this study were not consistent or convincing. The data presented here were collected as part of a multi-faceted study investigating the electroencephalographic (EEG) response to castration in *Bos indicus* bull calves during general anaesthesia [26]. In addition to the collection of EEG data, extensive behavioural analyses were undertaken before and after castration. 

An important feature of this study was that all of the animals underwent general anaesthesia as a part of the experimental design. This approach was necessary as the primary objective of the multi-faceted study, which this smaller study was a part of, was an investigation of EEG responses to castration. The assessment of post-operative pain is not possible with EEG and so other strategies were utilised for the two-week period following anaesthesia and surgery. The study design included a group that underwent anaesthesia without castration to control for the influence that anaesthesia had on the results. While the stress response to surgery and the extent to which anaesthetic techniques can modify it is well documented in humans, the stress response to anaesthesia (and the activities involved with anaesthesia such as transport, restraint and recovery) is poorly described [31]. The stress response to anaesthesia in cattle has been described with reference to changes in the pulse rate, respiratory rate, plasma cortisol and glucose concentrations, total plasma protein concentration, and haematocrit [32]. The anaesthetic regime in that study was based on xylazine or acepromazine for premedication prior to the administration of thiopentone, and it was concluded that anaesthesia was associated with an increase in pulse rate and haematocrit, and that recovery from anaesthesia was the most stressful period of short-term general anaesthesia [32]. The results of the current study cannot be compared, especially for the NC group, to that of Brearley et al. (1992) because of the entirely different anaesthetic drug regime and context [32]. Nevertheless, it is acknowledged that anaesthesia by any means will stimulate a stress response of some magnitude. In ponies, halothane-based anaesthesia without concomitant surgery was associated with a substantial adrenocortical response, suppression of insulin, and stress related metabolic changes [33]. With few exceptions, anaesthesia induces decreases in blood pressure, heart rate, and ventilation, along with alterations to the acid-base balance, but the extent to which these changes occur will vary with the anaesthetic drugs used. There are no data regarding the endocrine and metabolic impact of halothane anaesthesia on cattle and whether this procedure influences post-operative appetite, activity, and acute phase protein responses. It is possible that the consistent lack of difference between the treatment groups in the present study is attributable to anaesthesia. However, it is not possible to separate anaesthesia per se from the logistical aspects of the procedure, which include transport on the day of anaesthesia, isolation and restraint. The response to these stressors may have overshadowed the response to surgically induced pain. Prior habituation to the transport vehicle and the race and crush used for restraint prior to anaesthesia and surgery, may have enabled better differentiation between the effects of the procedure for anaesthesia and the pain associated with castration. 

General anaesthesia with halothane was necessary for the acquisition of the EEG in the animals in this study, and was performed as described by the “minimal anaesthesia model” [34,35]. Halothane is unlikely to provide any analgesia [36], however as halothane was used in each of the study groups any analgesic effects would be consistent in each group.

In this study, no difference in bodyweight was found between groups, which is consistent with other studies in *Bos indicus* [8,10] and in *Bos taurus* cattle [15]. A negative effect of a non-steroidal anti-inflammatory drug (NSAID) (ketoprofen) on bodyweight following castration has been reported, but the mechanism of this result is unclear [10]. Meloxicam is an NSAID, but the results of this study did not corroborate a potential negative effect on bodyweight in the meloxicam treated group, which presumably occurs due to a decrease in feed intake. Laurence et al. (2016) specifically investigated the effect of meloxicam administered post-operatively in *Bos indicus* cattle undergoing surgical castration and found that this approach to pain management following surgical castration had a positive effect on bodyweight [8]. These authors postulated that meloxicam offsets the negative welfare impact of surgical castration and increases the production because the animals are more likely to be active, exhibit normal reward seeking behaviour, and be more eager to walk to the feed trough and eat, than those animals feeling pain. Pelleted food was withheld prior to anaesthesia, and it is not known whether this could have created a situation where post-operative feed intake was determined more by hunger, instead of pain. 

Animals receiving meloxicam prior to castration in this study rested more often and for longer. This result contrasts with previous work, which demonstrated that acute post-operative pain was associated with more rest in *Bos taurus* and *Bos indicus* cattle [1,8,37]. There are a number of explanations for the disparity. First, an argument for resting behaviours can go either way. If animals that are in less pain are less restless and less active, it is feasible that reduced pain (associated with the administration of meloxicam) facilitates resting behaviour. This hypothesis is supported by the results of a study addressing pain following dehorning where calves receiving meloxicam were less active, but considered more comfortable, for the first five hours after the procedure [38]. The site of nociceptor activation may also be important and we speculate that the pain associated with dehorning may have less of an impact on resting than pain associated with castration, where a recumbent animal is resting on its site of operation. Conversely, animals may rest more often and for longer periods if they are in pain and reluctant to move. Second, the large variation in the results for each of the parameters employed in this study (activity, total rest time, and duration of rests) may indicate that the sample size was inadequate. Third, averaging of data over a 24-h period, instead of a shorter timeframe may have limited the identification of differences between the groups in the immediate post-operative period. Fourth, the overriding influence of anaesthesia on each study group is not completely understood and so its effect in this study was unknown. Finally, more specific parameters, such as average stride length, may be a more sensitive parameter to assess post-operative pain [37]. The pre-operative administration of the NSAID flunixin, in addition to a caudal epidural injection of lidocaine and epinephrine, minimised the decrease in stride length over the first 24 h following castration in young *Bos taurus* calves [37]. 

Pedometry holds promise as a pain assessment tool for cattle as it provides objective data (inferred as behavioural data), which, with careful interpretation with respect to the context, lends itself to statistical analyses. This tool, however, requires further investigation with a focus on averaging the results over shorter time periods (<24 h), consideration of more precise variables, such as stride length, and analysis in combination with behavioural ethograms.

In cattle, the increase in SAA and haptoglobin is expected to be major, and the increase in fibrinogen is likely to be moderate, following injury or infection [39,40,41,42]. It has therefore been presumed that the measurement of these APPs is useful for assessing stress associated with a painful procedure or a non-invasive stressful event like restraint and transportation. With a specific focus on the post-surgical acute phase response in horses, blood concentrations of the acute phase reactants SAA, fibrinogen, and iron correlated with the intensity of the surgical trauma [43]. Following a stressful experience of 4–6 h (transport and housing in solitary tie stalls), blood concentrations of SAA and haptoglobin increased in cattle for up to 48 h [40]. These two APPs increased in each of the study groups so that their relevance to pain following castration was difficult to determine in this context. These increases may be explained by the complex series of novel events that each animal experienced between days 0 and 3: segregation from the herd, a brief period of transport, restraint for induction of anaesthesia, at least 45 min of anaesthesia, recovery from anaesthesia, and return transport to the farm paddock. The increases in these markers in all of the study animals (including control animals) in the current study are consistent with other studies where cattle have been subjected to complex stress [40,44].

There are fewer data regarding the changes in blood concentrations of fibrinogen in response to stress in cattle, but it has been shown that psychological stress will cause an increase in this protein as well [18]. The increase in this APP was smallest in the NC group, which suggests that fibrinogen has potential to be used as a marker of an inflammatory response following surgery in *Bos indicus* bull calves. Although fibrinogen is considered a ‘moderate’ acute phase reactant, this conclusion is consistent with fibrinogen providing a substrate for fibrin formation during tissue repair following tissue trauma [41,45].

Iron is a constituent of serum and its decrease following cellular damage occurs due to changes in hepcidin expression. Hepcidin levels increase during inflammation and cause iron sequestration within macrophages and hepatocytes, along with decreased absorption of iron from the gastro-intestinal system. Analyses of blood iron concentrations in this study were unable to provide a means to differentiate treatment groups. However, the sampling frequency for this analyte may need to be different to the APPs that were also analysed in this study. The lowest blood concentration of iron occurred 24 h after surgery in horses, and returned to the baseline by 48 h [43]. It is unknown whether the temporal response in *Bos indicus* cattle is similar to horses. 

Future studies should consider more frequent blood sample collection than what was performed in this study, where the first post-operative sample was collected 3 days after surgery. The concentration of the APPs and iron are unknown between days 0 and 3 and given the potential for these values to both increase and decrease, or vice versa, in this timeframe, more frequent sampling should be performed in the future. Post-operative blood sampling on days 3 and 6 was performed in this study in an effort to strike a balance between the number of post-procedural interventions and useful data collection. This schedule may have prevented the identification of important alterations in the APPs and iron in the first one or days after surgery.

While interpreting the results of this study, there are a number of limitations that must be acknowledged. The impact of general anaesthesia, and the procedures associated with it, on the measured variables is largely unknown. The small sample size, especially of the NC group, make it difficult to address and separate the effects of anaesthesia alone on the responses of cattle after the procedures performed in this study. Furthermore, the size of the treatment groups (*n* = 2) was also relatively small. Pain is an experience with inherent individual variation, and a much larger sample size is required in the future. In a recently published meta-analysis of studies investigating changes in cortisol concentration, vocalization and average daily gain associated with castration in beef cattle, the authors concluded that results of multiple studies were inconclusive to draw recommendations on preferred castration practices to minimize pain in beef cattle [7]. Furthermore, the majority of studies included in the meta-analysis (13 of 22) reported a sample size ≤50. While this figure doesn’t confirm that “small” sample sizes are appropriate, it does reflect the logistic challenges of performing large studies with cattle. Interestingly, this meta-analysis also documents that in most studies the sample size is not justified.

## 5. Conclusions 

An interpretation of the results of this study is that the range of objective and quantifiable measurements that were employed were not useful for the assessment of acute pain in this cohort of animals. Growing bull calves in the novel environment of the University may not demonstrate consistent alterations in any of the measured parameters when they are subject to restraint stress, anaesthesia, and a painful procedure such as castration. If this interpretation is correct, alternative strategies to assess pain in this age group and species of animal are required. Nevertheless, the results of this study suggest that pre-operative administration of meloxicam is associated with animals being less restless after anaesthesia and surgery. The other objective assessments measured in this study (bodyweight, activity, and circulating acute phase protein and iron concentrations), were not able to consistently differentiate between treatment groups. These findings emphasise the need for alternative quantifiable and objective indicators of pain in *Bos indicus* bull calves.
